# Evaluation of Antibiotics Residues in Chicken Meat Samples in Lebanon

**DOI:** 10.3390/antibiotics8020069

**Published:** 2019-05-28

**Authors:** Adla Jammoul, Nada El Darra

**Affiliations:** 1 Lebanese Agricultural Research Institute, Food Department, Fanar P.O. Box: 2611, Lebanon; ajammoul@lari.gov.lb; 2 Beirut Arab University, Faculty of Heath Sciences, Tarik El Jedidah – Beirut, P.O. Box: 115020, Riad EL Solh 1107 2809, Lebanon

**Keywords:** chicken, antibiotic residues, HPLC, MRL

## Abstract

Antibiotic residue in chicken is a human health concern due to its harmful effects on consumer health. This study aims at screening the antibiotic residues from 80 chicken samples collected from farms located in different regions of Lebanon. An optimized multi-class method for identification and quantification of 30 antibiotics from four different chemical classes (sulfonamides, tetracyclines, quinolones, and beta-lactams) has been developed by using liquid chromatography–mass spectrometry. The evaluation of antibiotics residues in 80 chicken muscles samples has shown that 77.5% of samples were at least contaminated with antibiotics residues, out of which 53.75% were exposed to co-occurrence of multidrug residues. The screening of the four antibiotics families has shown that ciprofloxacin (quinolones) represents the highest occurrence percentage (32.5%), followed by amoxicillin (β-lactams) (22.5%) and then tetracyclines (17.5%). Means of sarafloxacin, amoxicillin, and penicillin G residues levels were above the Maximum Residue Limit (MRL) recommended limit according to the European Union EC. This study revealed that chicken samples collected from Lebanese farms contain antibiotic residues. Guidelines for prudent use of antimicrobials agents for chicken should be adopted to reduce the prevalence of resistant Salmonella in chicken.

## 1. Introduction

Over the last few decades, antibiotics have been widely used in animal husbandry, for their prophylactic and therapeutic purposes. It is to be noted that one-third of the antibiotics used in Europe are for veterinary application, with poultry and pigs livestock receiving the majority for their therapeutic purpose [1]. 

In poultry production, antibiotics are essential to prevent and control infectious diseases. They are also used illegally as feed supplements to stimulate animal growth and productivity [2,3]. The misuse and incorrect application of antibiotics carries the risk of their residues presence in edible tissues of the chicken, which can cause toxics and allergies in hypersensitive consumers [4]. Additionally, human exposure to high levels of antibiotics residues from animal sources may aggravate immunological response in low immune individuals and influence negatively the intestinal gut microbiota [5]. The misuse of antibiotics may trigger the development of resistant strains of bacteria, thus reducing the efficiency of antibiotics used for animal treatment, leading to the treatment failure of livestock, and affecting negatively the animal welfare [3]. The spread of antibiotic resistance is a problem of major concern globally. Therefore, the antibiotic residues are being considered as public health hazards [6], since there is a concern about the transfer of antibiotic-resistant genes from animal flora to human pathogens. Another risk comes from the transfer of antibiotic-resistant bacterial strains through the food chain [7].

To avoid exposing the end consumers to antibiotic residues risks, maximum residues limits (MRLs), in foodstuffs of animal origin, have been established by the European Commission and listed in the Commission Regulation (EU) 37/2010 [8].

Antibiotic residues in chicken meat samples have been reported by many research studies. Antibiotic residues were reported in broilers commercialized in Nigeria, with a high incidence rate (33.1%) [9]. The assessment of antibiotic residues in broiler chicken collected from farms located in Tanzania showed that 70% of the farms were positive to antimicrobial residues [10]. Another study conducted in Iran has screened enrofloxacin residue in chicken muscles collected from 90 broilers farms in Tahran. The study findings showed that 24% of the farms showed residues above the MRLs [11]. Furthermore, a research study, conducted in Turkey screening of quinolone antibiotics in 127 chicken samples, showed that 58 (45.7%) of chicken meat samples contain residues of quinolone [12].

According to the Lebanese Ministry of Agriculture data, there are about 115,000,000 chicken (a chicken with a weight of 1.9–2 kg produces about 1.1 kg of meat) that produce about 126,000 tons of meat per year. There are also small ventures in quail, duck, and turkey production. Exports are limited as most of the Arab states produce the major portion of their needs while imports are mainly from countries like Brasil, Thailand, and China where production costs are much lower than in Lebanon. The imported quantity last year was estimated at 8000 tons of poultry meat (less than 0.01% of our production). 

Many research studies, conducted in Lebanon, tested the presence of bacterial strains in the Lebanese livestock. The findings showed that Lebanese farms are potent reservoirs of multidrug-resistant bacteria, able to be transferred to humans [13,14,15]. A study conducted by Dandachi et al. in 2018, examining the prevalence of intestinal carriage of multidrug-resistant Gram-negative Bacilli in poultry farms at the national level, showed that that Lebanese poultry farms are potent reservoirs of antimicrobial resistance. The study emphasized on the importance on enforcing stricter rules to control the overuse and misuse of antibiotics in the poultry sector for treatment and growth promotion [16]. All the research studies conducted in Lebanon asked for data about the antibiotic residues in poultry. 

To the best of our knowledge, major gaps were found at the study level of antibiotics residues occurrence in chicken samples collected from Lebanese farms.

The main purpose of this study is to monitor the incidence of antibacterial residues in chicken meat samples (80) collected from Lebanese farms, to produce occurrence data to be used for the exposure assessment to these residues. The samples have been analyzed for 30 antibiotics compounds from the four families, sulfonamides, quinolones, tetracyclines, and beta-lactam antibiotics. The samples were extracted through a modified QuEChERS extraction/cleanup method and then analyzed by using LC–MS/MS method. The validation was done according to the directives of the European Commission Decision 2002/657/EC [17]. This study will provide information on the possible multi-occurrence of antibiotic residues in chicken samples commercialized in Lebanon. This research evaluated the northern area of Lebanon, from the capital to the north.

## 2. Materials and Methods

### 2.1. Sampling

A total of 80 chicken samples (fresh broilers) were randomly collected from one slaughterhouse located in Keserwan, in the Mount Lebanon Governorate. The 80 samples in this slaughterhouse came from 19 farms located in different regions of Lebanon. The 19 farms were distributed as follows. Four farms (providing 4 random samples each) were located in Jbeil, the Mount Lebanon Governorate, four farms (providing 4 random samples each) were located in Keserwen, the Mount Lebanon Governorate, four farms (providing 4 random samples each) were located in Batroun, North Lebanon, four farms (3 farms providing 4 random samples and one farm providing 5 samples each) were located in Akkar, North Lebanon, and three farms (providing 5 random samples each) were located in Metn, the Mount Lebanon Governorate. 

Each sample was placed into a separate plastic zipper bag, numbered and then transferred to the laboratory in an ice plastic container and stored at −20 °C until extraction. These samples were subjected later to grinding before the extraction procedure.

### 2.2. Chemicals and Reagents

All reagents were of analytical grade. Antibiotic standards of 4 different families, sulfonamides (sulfacetamide, sulfapyridine, sulfamerazine, sulfamethazine, sulfadimidine, sulfameter; sulfamethoxypyridazine, sulfachloropyridazine, sulfadimethoxine, sulfadoxine, sulfamethoxazole, sulfabenzamide, and sulfaquinoxaline), quinolones (nalidixic acid, oxolinic acid, ciprofloxacin, and norfloxacin, ofloxacin, flumequine, danofloxacin, enrofloxacin, lomefloxacin, and sarafloxacin), tetracyclines (tetracycline, oxytetracycline, and doxycycline), and beta-lactam antibiotics (amoxicillin, ampicillin, cloxacillin, and penicillin G), were purchased from (Sigma-Aldrich, St louis, MO, United States). All the standards were of high purity grades (>99%). Individual stock solutions were prepared at 1 g/L in acetonitrile and stored at −18 °C.

The working standard solutions containing all analytes except the lactams group with variable concentrations, according to their MRLs, was prepared as dilution of the stock solution in water /acetonitrile ratio of 70:30 (*v/v*), and the working standard solutions were kept at −20 °C in brown glass and were used for 1 week. 

HPLC-grade water, HPLC-grade acetonitrile, trifluoroacetic acid (TFA; 0.2%), disodium ethylenediaminetetraacetate dihydrate (Na_2_EDTA), and magnesium sulfate (MgSO_4_) were also supplied by Sigma-Aldrich. 

### 2.3. Determination of Antibiotics Residues 

#### 2.3.1. Sample Extraction

A multi-class method for identification and quantification of 30 antibiotics from four different chemical classes (sulfonamides, tetracyclines, quinolones, and beta lactams) has been developed by using liquid chromatography–mass spectrometry. The method was optimized for detection of antibiotics in muscles of chicken meat. The optimized method was validated according to the European Commission Directive 2002/657/EC [17]. Chicken muscle meats of around 500 g were homogenized in a laboratory blender, to increase the detection change of drug residues. Afterwards, 4 g of minced chicken muscle was weighed in a 20 mL glass centrifuge tube. Four milliliters of ultrapure water was added, then vortexed for 60 s and kept in the dark for 10 min.

Then, 1 mL of EDTA was added as a chelating agent, to compete with antibiotics as tetracyclines. This compound improved as well the performance of these antibiotics and avoid their losses [18]. Ten milliliters of acetic acid (1%) in acetonitrile was added, and then shaken vigorously for 1 min. Next, magnesium sulfate (MgSO_4_; 4 g) and sodium chloride (NaCl: 1 g) were added. The tubes were subjected to centrifugation at 5000 rpm at 4 °C for 5 min and then a resting for 30 min.

Six milliliters of the supernatant was mixed with 50 mg of primary secondary amine (PSA), 150 mg C18, and 900 mg of MgSO_4_. Another centrifugation was conducted at 5000 rpm at 4 °C for 5 min. Four milliliters of the supernatant obtained was transferred to a new tube and evaporated under a gentle stream of nitrogen at 50 °C. The residue was redissolved in 1 mL solution with a mobile-phase water/acetonitrile ratio (70:30, *v/v*), and then subjected to filtration through a 0.45 μm polyvinylidene fluoride (PVDF) filter for further LC–MS/MS analysis under Multiple Reaction Monitoring MRM-optimized conditions for each compound (Table 1), which is a quick, easy, cheap, effective, rugged, and safe methodology introduced in 2003 by Anastassiades et al. [19].

#### 2.3.2. LC–MS/MS Equipment

LC–MS/MS analyses were performed on a triple quadrupole tandem Agilent 6430 LC/MS system (California, USA). The positive electrospray ion source (ESI+) was used with data acquisition in multiple reaction monitoring (MRM) mode (ionspray voltage: 4 kV, nitrogen for desolvation and dried gas: 11 L/min.

Quantification of the four antibiotics families in 80 poultry samples was performed by measuring peak areas in the MRM chromatogram and comparing them with the relevant matrix-matched calibration curves. A calibration curve ranged between 5 and 200 μg/L was done to verify the linearity. 

#### 2.3.3. LC–MS/MS Parameters 

The separation of the antibiotic residues was performed using a C18 analytical column (zorbax 2.1 mm inner diamater I.D × 150 mm length, 3.5 μm particle size; California, USA). The separation of beta–lactam was accomplished at 30 °C. The flow rate and injection volume were 0.5 mL/min and 10 µL, respectively. The mobile phases used were (A) water and (B) acetonitrile. The gradient elution program started with 10% B for 1 min, increased to 65% for 6 min, then increased to 95% for 1 min and returned to the initial conditions in 1 min. The final run time of the method was 12 min. 

The separation of sulfamides, tetracyclines, and quinolones was accomplished at 40 °C. The flow rate and injection volume were 0.3 mL/min and 10 µL, respectively. The mobile phases used were (A) TFA (0.1%) in water and (B) acetonitrile. The gradient elution program was as follow: A (90%) (3 min), A (25%) (5 min), and A (90%) (1min); the final run time of the method was 15 min. 

#### 2.3.4. Recovery Test

In-house validation was performed by fortifying the blank matrix at three levels 0.5, 1.0, and 1.5 MRL in triplicate, respectively, or at concentrations as low as possible for substances without an MRL. The extraction was performed by the methods described in Section 2.3.1. The spiked and blank samples were then analyzed by LC–MS/MS. Recovery was calculated by comparing the analyzed concentrations with spiked concentrations [20].

## 3. Results and Discussion

### 3.1. Method Performance 

The method was validated in-house according to the criteria specified in EU Commission Decision 2002/675/EC [17] for a quantitative method, and the validation parameters were determined by spiking blank chicken meat at three levels 0.5, 1, and 1.5 MRL. The measured parameters were specificity, linear range, repeatability, reproducibility, accuracy, and limit of quantification (LOQ). 

The performance of the analytical method was evaluated by checking the identification criteria for the presence of two transitions at the same retention time, the signal-to-noise ratio of ≥10, the relative retention time of the analyte within a tolerance of 2.5%, and the relative ion intensities ratio within a tolerance defined by the EU Commission Decision 2002/657/EC. The calculated ion ratios for the matrix are shown in Table 1.

**Figure 1:**
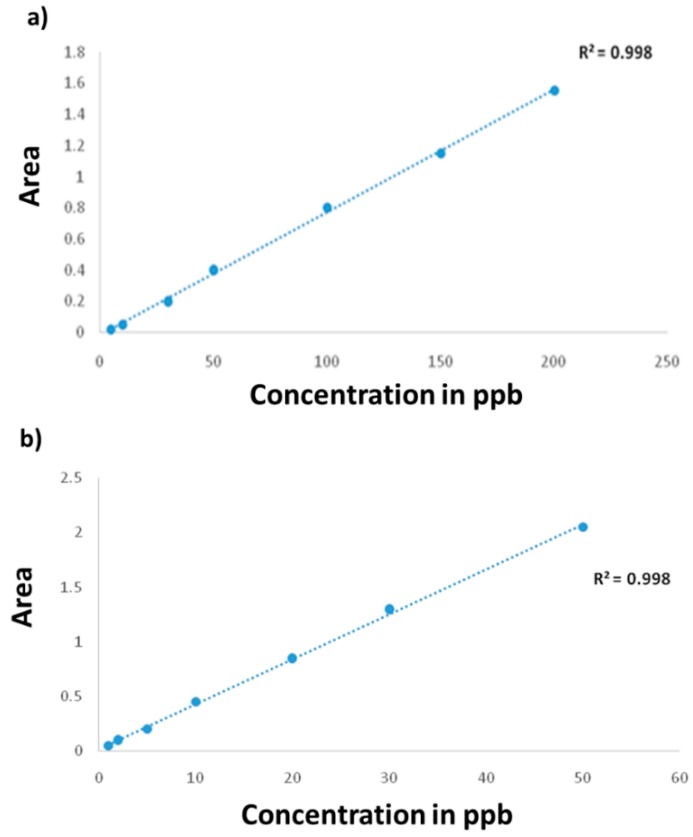

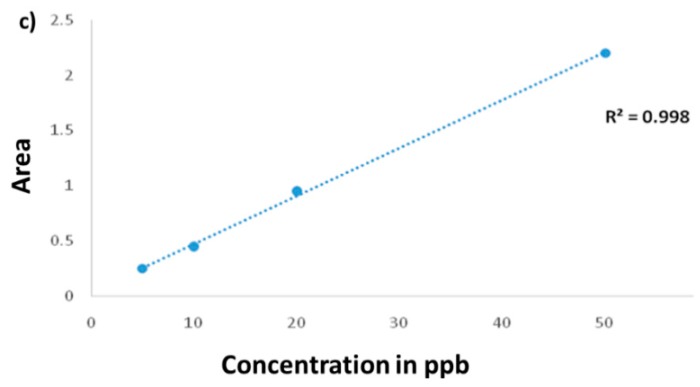
HPLC standard calibration curves for (**a**) ampicillin standards at 50, 100, 150, 200, and 250 ppb; (**b**) nalidixic acid at 1, 5, 10, 20, 30, and 50 ppb; (**c**) sulfadoxine at 5, 10, 20, and 50 ppb.

The chromatograms of the reference standards and calibration curves of all the antibiotic residues are shown in Figure 1. The calibration curves were created from 6 matrix-calibration standards which were injected in each batch in the range of 5 to 200 ppb. The calibration curves showed good linearity, characterized by a high correlation coefficient (r^2^ > 0.99). 

The precision of the method was determined using the spiked samples at three levels. In the same day, the set of samples was measured at 3 replicates. The results for repeatability ranged from 0.6% to 18.8% (Table 2).

The LOQ was considered as the lowest quantified level with a Signal/ Noise ratio (S/N ratio of ≥10) in the presence of the two transitions at the same retention time. The LOQs were calculated between 5 and 10 μg/kg for all tested antibiotics (Table 2). 

Out of the 30 antibiotics tested for their recoveries at three different levels, 24 antibiotics presented high mean recoveries, and the other 6 antibiotics presented lower mean recoveries, which were still higher than 50%. The mean recoveries of the residues for the spiked samples ranged between 53% and 110.5% (Table 2). These values were within the acceptable ranges (50–120%) recommended by [21].

### 3.2. Occurrence of Antibiotic Residues in Chicken Meat Samples

The method developed was applied to the determination of 30 antibiotics from four different chemical classes (sulfonamides, quinolones tetracyclines, and beta-lactams) in eighty chicken samples collected from a slaughterhouse originating from several farms of Lebanon. In order to validate the results, an internal quality control was carried out on every batch of samples. Moreover, the retention time, quantification and confirmation transitions, and relative ion intensities of the detected ion in chicken samples were compared to those of the corresponding calibration standards in the same batch to identify the detected analytes using the criteria established by [17]. Violative samples were defined as those ones exceeding the substance MRL according to EU relevant legislation [17].

While assessing the antibiotic residues in the chicken samples (Table 3), the results showed that none of the 30 antibiotics were detected in 22.5% of samples. 77.5% of samples were contaminated at least with one residue. Out of the contaminated samples, 23.75% were contaminated with one antibiotic residue and 53.75% were contaminated with more than one antibiotic residue. This level of contamination is higher than the incidence found in a study conducted in Portugal on 92 samples of chicken muscles for four fluoroquinones, of which the contamination level was found to be only 42% [22]. This could be probably due to the misuse of antibiotics in food animal production in the Lebanese farms, together with the violation of the withdrawal period regulation [23]. 

### 3.3. Mean Concentrations of Antibiotic Residues for the Different Families in Chicken Samples

Table 4 represents the summary of multi-antibiotic residues occurrence in the chicken samples. The assessment of the sulfonamides family (Table 4) showed that 6 antibiotic residues were not detected in all the chicken samples, sulfachloropyridazine, sulfameter, sulfapyridine, sulfadoxine, sulfamethoxypyridazine, and sulfamerazine. However, the others (7) were detected with a percentage of positive samples ranging from 1.25% to 3.75%. It is to be noted that sulfonamides were the family less detected than the other families. For the sulfonamides, all the mean values were acceptable and did not exceed the MRL (100 μg/kg) according to the European Commission EC (2010) [8]. This result is in correlation with a study conducted in Malaysia aiming to screen chicken samples collected from 11 different states. The results showed that the mean values of sulphonamides in all the chicken samples were below the MRLs established in Malaysia (0.1 µg/g) [24].

While assessing the quinolones (Table 5), the percentage of positive samples ranged from 1.25% to 32.5%, with the highest percentage obtained among all the antibiotics residues tested for ciprofloxacin (32.5%). However, the ciprofloxacin mean level did not exceed the MRL (100 μg/kg). It is to be noted that all the positively contaminated chicken samples did not exceed the MRLs for the all the quinolones residues except for sarafloxacin, with a value of 19 μg/kg, almost double the MRL (10 μg/kg) according to the European Union EC (2010) [8]. Many studies commonly reported the presence of quinolones in chicken with a mean value of 30 μg/kg, similar to the one obtained in our study [25,26]. These studies have shown that the introduction of quinolone usage in poultry farms has led to the development of resistant strains of Salmonella sp. and Campylobacter jejuni, isolated from poultry meat. Thus, our study confirms the misuse of quinolones in Lebanese farms noted by lack of implementation of recommended withdrawal times. Consequently, there is a need for a serious intervention. 

The assessment of the tetracyclines family (Table 6) showed a percentage of positive samples ranging from 3.75% to 17.5%, with tetracycline presenting the highest mean level among tetracyclines. All the samples did not exceed the MRL for tetracyclines (100 μg/kg). However, importance should be given to the presence of tetracycline since a study conducted on Salmonella isolated from poultry products in the city of Porto showed a high resistance percentage of 36% for tetracyclines [27]. A control on the tetracycline usage should be adopted in Lebanon. 

While assessing the β-lactams (Table 7), the percentage of positive samples ranged from 5% to 22.5%, with the highest percentage (22.5%) noted for amoxicillin and the lowest (5%) for cloxacillin. Amoxicillin was found to be the second highest residue found in the chicken samples, after ciprofloxacin. It is to be noted that all the positives samples for ampicillin and cloxacillin did not exceed the MRL according to the European Union EC (2010) [8], which are 50 and 300 μg/kg, respectively, and 18.7% of the samples were shown positive to ampicillin. This result could be linked to the study conducted in Lebanon assessing the multidrug-resistant bacteria isolated from Lebanese farms. Out of 235 bacterial strains, isolated from 981 fecal swabs from 49 Lebanese poultry farms, 92% were for *E. coli* presenting high resistance for ampicillin [16].

However, 3 chicken samples out of 80 were contaminated with mean values of amoxicillin (63, 62.5, and 77.5 μg/kg), exceeding the MRL (50 μg/kg). Moreover, 4 samples were contaminated with mean values of penicillin G (55, 72.5, 133, and 198 μg/kg) exceeding the MRL (50 μg/kg). This could be linked to inappropriate use of antimicrobial drugs in food-producing animals. As a consequence of exceeding MRLs, toxicological effects and allergic reactions can reach the consumers of the chicken [28]. The high mean values of penicillin G are alarming since recent research studies have shown that staphylococcus aureus isolated from 150 samples of chicken and raw meat present a high prevention of penicillin G resistance (53.8%) [29]. More restrictive policies on the use on Penicillin G in animal husbandry may improve the current situation.

Sulfonamides represented the lowest percentage of positive samples. While comparing the other families, we found that ciprofloxacin (quinolones) represents the highest percentage (32.5%), followed by amoxicillin (β-lactams) (22.5%) and then tetracyclines (17.5%). These findings are in accordance with a study conducted in Bangladesh on 200 samples of chicken muscles showing similar percentages for ciprofloxacin (34%), amoxicillin (22%), and tetracyclines (20%) [24]. This could be due to the fact that tetracyclines and amoxicillin are among the most commonly used antibiotics in animal husbandry [30].

The results of our studies are of interest since food-producing animals were shown to be a potent reservoir of multidrug-resistant organisms [31]. Thus, the high prevalence of antibiotic residues could be a source of resistance developments among many bacterial strains. To decrease the resistance rate of bacteria, monitoring of resistance, surveillance, prudent use, research projects, awareness, and educational programs are recommended by WHO [32].

## 4. Conclusions

This preliminary research was reported for the first time, to the best of our knowledge, the presence of antibiotic residues in poultry samples coming from Lebanese farms. The screening of the 80 samples of chicken presents a co-occurrence of multidrug residues in 53% of the samples, posing consequently serious public health concerns to humans and animals, such as toxicity, allergic reactions, and resistance development. The level of antibiotic consumption in the Lebanese veterinary medicine should be evaluated. This study urges the need of an intervention to decrease the level of antibiotics residues in chicken samples through launching educational and awareness programs on the prudent use of antibiotics in animal husbandry. This study emphasized on the importance of respecting the withdrawal periods of antimicrobials, aiming to reduce the level of antimicrobial residues in chicken samples to a minimum and to stress on controlling the farms through regular sampling and analysis. Further studies should be conducted to screen a larger sample collected from different farms located in different regions in Lebanon. The subsequent researches would need focus on the southern part of Lebanon to complete the available data and to provide a complete view of antibiotic residues in chickens for entire country.

## Figures and Tables

**Table 1:** Multiple reaction monitoring (MRM) acquisition condition for each antibiotic used.

Antibiotics		Precursor Ion (m/z)	Product Ion (m/z)	Cone Voltage (V)	Collision Energy (eV)	Retention Time (min)
Sulfonamides	Sulfacetamide	215	156, 92	75	4, 24	5.66
	sulfapyridine	250.1	156, 92	110	16, 36	6.27
	Sulfamerazine	265.1	92, 156	110	32, 16	6.46
	Sulfamethazine	279.1	124, 186	110	24, 16	6.99
	Sulfadimidine	279.1	124, 186	105	24, 16	7.00
	Sulfameter	281.1	92, 156	115	32, 16	7.76
	Sulfamethoxypyridazine	281.1	116, 92	120	16, 32	8.05
	Sulfachloropyridazine	285	156, 92	100	12, 28	9.17
	Sulfadimethoxine	311.1	156, 92	125	20, 36	9.86
	Sulfadoxine	311.1	156, 92	115	16, 32	13.83
	Sulfamethoxazole	254.1	156, 92	90	12, 28	10.25
	Sulfabenzamide	277.1	92, 156	90	32, 8	13.15
	Sulfaquinoxaline	301.1	156, 92	120	16, 36	14.23
Quinolones	Nalidizic acid	233.1	251, 187	85	12, 28	7.14
	Oxolinic acid	262.1	244, 202	100	16,36	7.50
	Ciprofloxacin	332.1	314, 231	125	20,40	4.62
	Norfloxacin	320.1	302, 198.5	125	20,44	4.50
	Ofloxacin	362.2	344, 318	135	20,20	4.50
	Flumequine	262.1	244, 202	110	16, 36	6.00
	Danofloxacin	358.2	340, 82	140	24, 52	4.78
	Enrofloxacin	360.2	342, 316	115	20, 20	5.04
	Lomefloxacin	352.2	334, 265	120	20, 24	4.76
	Sarafloxacin	386.1	368, 342	135	20, 16	5.56
Tetracyclines	Tetracycline	445.2	428, 154	110	16, 40	5.67
	Oxytetracycline	461.2	443, 426	130	10, 16	5.55
	Doxycycline	445.2	410, 154	125	16, 22	5.97
β-lactams	Amoxicillin	366.1	348.9, 114	98	4, 16	0.925
	Ampicillin	350.1	174, 106.1	98	12, 16	1.830
	Cloxacillin	436.1	160, 114.1	98	8, 44	6.10
	Penicillin G	335.1	176, 160	75	12, 8	5.38

**Table 2:** Results of in-house validation of the LC–MS/MS method for the antibiotics considered in this study, with the maximum residue limits (MRLs) established by the European Union (2010) [8]. STDEV: standard deviation. RSDr: relative standard deviation of repeatability. LOQ: limit of quantification.

Antibiotics	Mean Recoveries (%) of 50, 100, 150 (µg/kg)	STDEV	RSDr (%)	LOQ (µg/kg)	MRL μg/kg or ppb in Muscles EC, (2010)
Sulfonamides					
Sulfacetamide	65.2, 64.89, 71.17	2.5, 0.7, 3.36	7.9, 1.09, 3.14	10	100
Sulfapyridine	53.76, 64.7, 66.83	0.29, 0.7, 2.35	1.07, 1.08, 2.34	10	100
Sulfamethazine	93, 81.34, 86.34	1.93, 1.55, 2.63	4.15, 1.9, 2.03	5	100
Sulfadimidine,	86.87, 75.4, 78.71	1.16, 0.8, 0,73	2.68, 1.06, 0.61	5	100
Sulfamethoxypyridazine	82.39, 69.13, 70.04	0.8, 1.22, 3.8	1.95, 1.77, 3.6	10	100
Sulfachloropyridazine	90.71, 84.59, 96.37	0.93, 0.81, 3.37	2.05, 0.96, 2.33	5	100
Sulfadimethoxine	70.96, 87.9, 85.83	6.67, 16.03, 12.82	18.8, 18.24, 9.96	10	100
Sulfadoxine	68.73, 95.1, 84.01	4.395, 12.5, 15.32	12.78, 13.14, 12.15	5	100
Sulfamethoxazole	75.92, 92.26, 110.5	0.68, 10.73, 19.75	1.8, 11.63, 11.91	5	100
Sulfabenzamide	61.39, 81.49, 90.76	4.01, 1.35, 6.6	13.07, 1.66, 4.85	10	100
Sulfaquinoxaline	66.85, 79, 81.93	2.175, 7.6, 17.5	6.5, 9.6, 14.23	5	100
Quinolones					
Ciprofloxacin	62.33, 80.01, 84.46	0.92, 7.68, 6.19	2.96, 9.6, 4.89	10	100
Sarafloxacin	61.58, 74.93, 82.86	1.47, 4.04, 6.39	4.77, 5.39, 5.14	10	10
Danofloxacin	60.14, 79.66, 87.82	0.88, 1.11, 12.82	2.94, 1.39, 9.73	10	200
Tetracyclines					
Tetracycline	72.192, 78.34, 88.14	2.62, 4.54, 2.21	7.26, 5.8, 1.67	5	100
Oxytetracycline	101.25, 76.5, 96.07	8.88, 1.36, 2.98	17.54, 1.78, 2.07	5	100
β-lactams					
Penicillin G	61.82, 71.3, 74.43	2.93, 2.81, 3.52	18.99,7.89, 6.31	10	50

**Table 3:** Occurrence of antibiotic residues in chicken meat samples.

	Contaminated Samples (77.5%)		Non-Contaminated
	Mono-Contaminated	Poly-Contaminated	
Chicken samples (*n* = 80)	19 (23.75%)	43 (53.75%)	18 (22.5%)

**Table 4:** Occurrence in the 80 chickens samples of sulfonamides.

Chicken Samples (*n* = 80)	Sulface-Tamide	Sulfame-Thazine	Sulfadi-Midine	Sulfadime-Thoxine	Sulfame-Thoxazole	Sulfaben-Zamide	Sulfaqui-Noxaline
mean (µg/kg)	0.25	0.2	0.4	0.3	0.06	0.3	0.1
min (µg/kg)	nd	nd	nd	nd	nd	nd	nd
max (µg/kg)	10	17.3	11.7	14.8	3	11.2	11.8
*n* positive	2	1	3	2	2	3	1
% positive	2.5	1.25	3.75	2.5	2.5	3.75	1.25

**Table 5:** Occurrence in the 80 chickens samples of quinolones.

Chicken Samples (*n* = 80)	Nalidixic Acid	Oxolinic Acid	Ciprofl-Oxacin	Norfl-Oxacin	Ofl-Oxacin	Flume-Quine	Danofl-Oxacin	Enrofl-oxacin	Lomefl-Oxacin	Sarafl-Oxacin
mean (µg/kg)	0.1	0.1	6.2	0.7	2.28	0.1	0.3	1.6	0.6	0.4
min (µg/kg)	nd	nd	nd	nd	nd	nd	Nd	nd	nd	nd
max (µg/kg)	14.6	10	32.5	18.7	24.3	10	9.4	27.7	9.6	19
*n* positive	8	1	26	4	15	1	4	10	6	3
% positive	10	1.25	32.5	5	18.75	1.25	5	12.5	7.5	3.75

nd: not determined.

**Table 6:** Occurrence in the 80 chickens samples of tetracyclines.

Chicken Samples (*n* = 80)	Tetracycline	Oxytetracycline	Doxycycline
mean (µg/kg)	24.4	22.6	11.4
min (µg/kg)	8.4	9.6	8.6
max (µg/kg)	63.8	46.2	15.6
*n* positive	14	8	3
% positive	17.5	10	3.75

nd: not determined.

**Table 7:** Occurrence in the 80 chickens samples of β-lactams.

Chicken Samples (*n* = 80)	Amoxicillin	Ampicillin	Cloxacillin	Penicillin G
mean (µg/kg)	5.4	1	0.3	8.5
min (µg/kg)	nd	nd	nd	nd
max (µg/kg)	77.5	22.6	14.3	198
*n* positive	18	15	4	13
% positive	22.5	18.7	5	16.2

nd: not determined.

## References

[1] Thiele-Bruhn S. (2003). Pharmaceutical antibiotic compounds in soils - A review. J Plant Nutr. Soil.

[2] Kantiani L., Farré M., Grases i Freixiedas J.M., Barceló D. (2010). Development and validation of a pressurised liquid extraction liquid chromatography-electrospray-tandem mass spectrometry method for β-lactams and sulfonamides in animal feed. J. Chromatogr. A.

[3] Laxminarayan R., Duse A., Wattal C., Zaidi A.K., Wertheim H.F., Sumpradit N., Vlieghe E., Hara G.L., Gould I.M., Goossens H. (2013). Antibiotic resistance-the need for global solutions. Lancet Infect. Dis..

[4] Marazuela M.D., Bogialli S. (2009). A review of novel strategies of sample preparation for the determination of antibacterial residues in foodstuffs using liquid chromatography-based analytical methods. Anal. Chim. Acta.

[5] Normanno G., La Salandra G., Dambrosio A., Quaglia N.C., Corrente M., Parisi A., Santagada G., Firinu A., Crisetti E., Celano G.V. (2007). Occurrence, characterization and antimicrobial resistance of enterotoxigenic Staphylococcus aureus isolated from meat and dairy products. Int. J. Food Microbiol..

[6] Haller M.Y., Müller S.R., McArdell C.S., Alder A.C., Suter M.J.F. (2002). Quantification of veterinary antibiotics (sulfonamides and trimethoprim) in animal manure by liquid chromatography-mass spectrometry. J. Chromatogr. A.

[7] Barton M.D. (2005). Antibiotic use in animal feed and its impact on human healt. Nutr. Res. Rev..

[8] Commission E. (2010). Euopean Commission Regulation Commission Regulation (EU) No 37/2010 of 22 December 2009 on pharmacologically active substances and their classification regarding maximum residue limits in foodstuffs of animal origin.

[9] Kabir J., Umoh V.J., Audu-okoh E., Umoh J.U., Kwaga J.K.P. (2004). Veterinary drug use in poultry farms and determination of antimicrobial drug residues in commercial eggs and slaughtered chicken in Kaduna State, Nigeria. Food Control.

[10] Nonga H.E., Mariki M., Karimuribo E.D., Mdegela R.H. (2009). Assessment of antimicrobial usage and antimicrobial residues in broiler chickens in Morogoro Municipality, Tanzania. Pakistan J. Nutr..

[11] Salehzadeh F., Salehzadeh A., Rokni N., Madani R., Golchinefar F. (2007). Enrofloxacin residue in chicken tissues from Tehran slaughterhouses in Iran. Pakistan J. Nutr..

[12] Er B., Kaynak Onurdǎ F., Demirhan B., Özgen Özgacar S., Bayhan Öktem A., Abbasoǧlu U. (2013). Screening of quinolone antibiotic residues in chicken meat and beef sold in the markets of Ankara, Turkey. Poult. Sci..

[13] Al Bayssari C., Dabboussi F., Hamze M., Rolain J.-M. (2014). Emergence of carbapenemase-producing Pseudomonas aeruginosa and Acinetobacter baumannii in livestock animals in Lebanon. J. Antimicrob. Chemother..

[14] Rafei R., Hamze M., Pailhoriès H., Eveillard M., Marsollier L., Joly-Guillou M.-L., Dabboussi F., Kempf M. (2015). Extrahuman epidemiology of Acinetobacter baumannii in Lebanon. Appl. Environ. Microbiol..

[15] Diab M., Hamze M., Madec J.-Y., Haenni M. (2017). High prevalence of non-ST131 CTX-M-15-producing Escherichia coli in healthy cattle in Lebanon. Microb. Drug Resist..

[16] Dandachi I., Sokhn E.S., Dahdouh E.A., Azar E., El-Bazzal B., Rolain J.M., Daoud Z. (2018). Prevalence and characterization of multi-drug-resistant gram-negative bacilli isolated from lebanese poultry: A nationwide study. Front Microbiol..

[17] Commission E. (2002). European Commission Decision of 12 August 2002 Implementing Council Directive 96/23/EC Concerning the Performance of Analytical Methods and the Interpretation of Results.

[18] Freitas A., Barbosa J., Ramos F. (2014). Multi-residue and multi-class method for the determination of antibiotics in bovine muscle by ultra-high-performance liquid chromatography tandem mass spectrometry. Meat Sci..

[19] Anastassiades M., Lehotay S.J., Stajbaher D., Schenck F.J. (2003). Fast and Easy Multiresidue Method Employing Acetonitrile. J. AOAC Int..

[20] Ramatla T., Ngoma L., Adetunji M., Mwanza M. (2017). Evaluation of Antibiotic Residues in Raw Meat Using Different Analytical Methods. Antibiotics.

[21] Association of Official Analytical Chemists (2002). AOAC Guidelines for Single Laboratory Validation of Chemical Methods for Dietary Supplements and Botanicals.

[22] Pena A., Silva L.J.G., Pereira A., Meisel L., Lino C.M. (2010). Determination of fluoroquinolone residues in poultry muscle in Portugal. Anal. Bioanal. Chem..

[23] Sattar S., Hassan M.M., Islam S.K.M.A., Alam M., Al Faruk M.S., Chowdhury S., Saifuddin A.K.M. (2014). Antibiotic residues in broiler and layer meat in Chittagong district of Bangladesh. Veterinary World.

[24] Cheong C.K., Hajeb P., Jinap S., Ismail-Fitry M.R. (2010). Sulfonamides determination in chicken meat products from Malaysia. Int. Food Res. J..

[25] Kim D.P., Degand G., Douny C., Pierret G., Delahaut P., Ton V.D., Granier B., Scippo M.-L. (2013). Preliminary Evaluation of Antimicrobial Residue Levels in Marketed Pork and Chicken Meat in the Red River Delta Region of Vietnam. Food Public Health.

[26] Jayalakshmi K., Paramasivam M., Sasikala M., Tamilam T.V., Sumithra A. (2017). Review on antibiotic residues in animal products and its impact on environments and human health. J. Entomol. Zool. Stu..

[27] Antunes P., Réu C., Sousa J.C., Peixe L., Pestana N. (2003). Incidence of Salmonella from poultry products and their susceptibility to antimicrobial agents. Int. J. Food Microbiol..

[28] Demoly P., Romano A. (2005). Update on beta-lactam allergy diagnosis. Curr. Allergy Asthma Rep..

[29] Gundogan N., Citak S., Yucel N., Devren A. (2005). A note on the incidence and antibiotic resistance of Staphylococcus aureus isolated from meat and chicken samples. Meat Sci..

[30] Mayrhofer S., Paulsen P., Smulders F.J.M., Hilbert F. (2004). Antimicrobial resistance profile of five major food-borne pathogens isolated from beef, pork and poultry. Int. J. Food Microbiol..

[31] Bachiri T., Bakour S., Ladjouzi R., Thongpan L., Rolain J.M., Touati A. (2017). High rates of CTX-M-15-producing Escherichia coli and Klebsiella pneumoniae in wild boars and Barbary macaques in Algeria. J. Glob. Antimicrob. Resist..

[32] World Health Organization (2001). Antibiotic Resistance: Synthesis of Recommendations by Expert Policy Groups. Alliance for the Prudent Use of Antibiotics.

